# Supplementary figures and images for: An efficient propagation system through stem cuttings of a multipurpose plant—Ficus tikoua Bur
Source: PeerJ. 2024 Dec 24;12:e18768. doi: 10.7717/peerj.18768 (PMC11674247; doi:10.7717/peerj.18768)

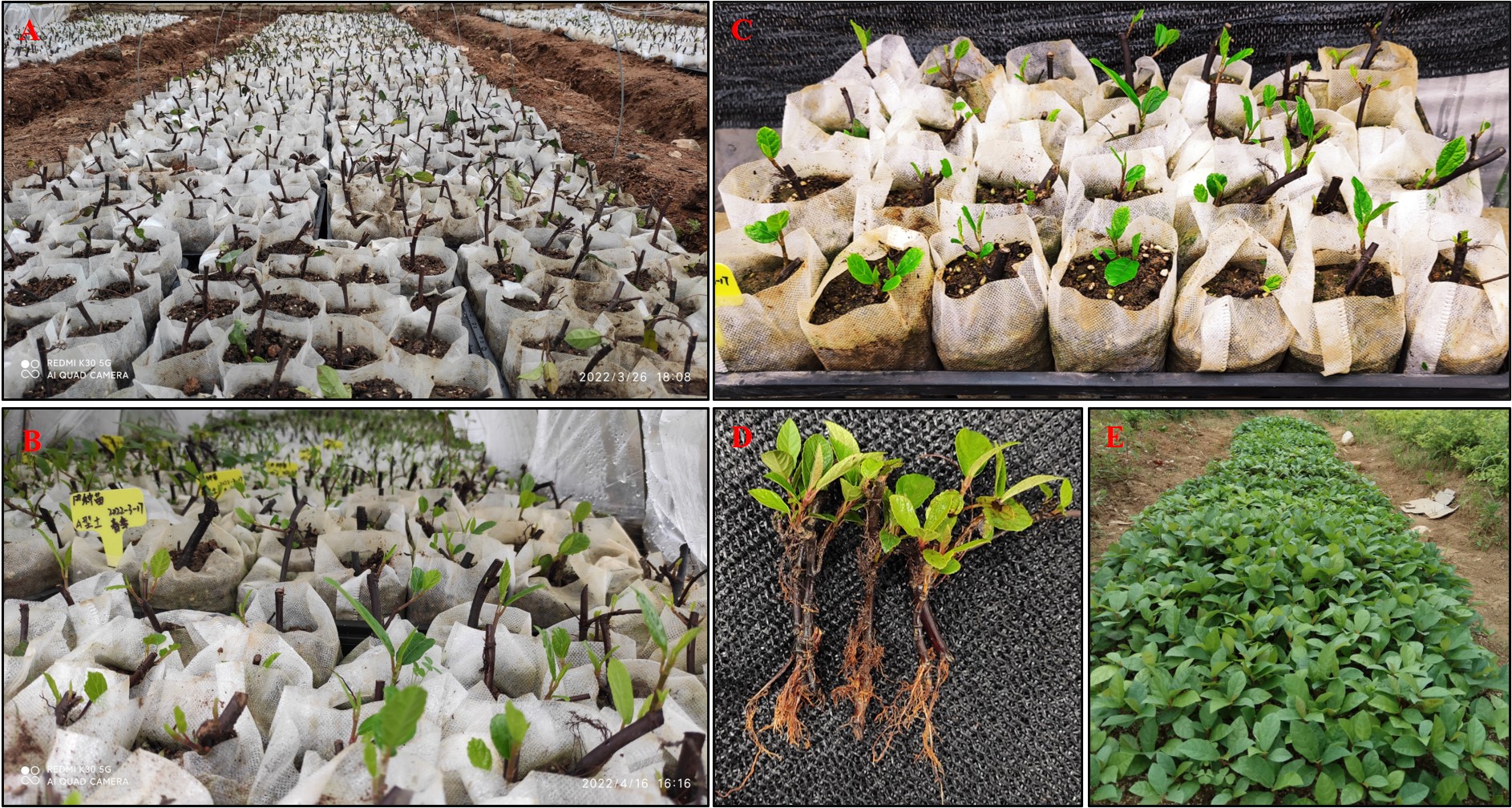

Supplement: Figure S1 — (A) Initial Phase of Stem Cutting Propagation: This image depicts the early stage following the insertion of stem cuttings into the propagation medium, showcasing the initial conditions before any growth is observed.(B) Emergence of Adventitious Buds: Illustrates the critical phase where adventitious buds begin to form along the stem cuttings, indicating the commencement of the vegetative growth process.(C) Bud Development and Elongation: Further development of the adventitious buds into visible elongated structures, highlighting the progress of the propagation process.(D) Maturation into Fully Developed Seedlings: Represents the successful culmination of the propagation process, where the stem cuttings have developed into robust, fully formed seedlings ready for transplanting or further growth.(E) Late-Stage Seedling Growth and Vigor: Captures the health and vitality of the seedlings as they enter the final phase of propagation, demonstrating the overall success and efficiency of the propagation technique used. [file peerj-12-18768-s002.jpg]
